# Comparison of dry and wet electroencephalography for the assessment of cognitive evoked potentials and sensor-level connectivity

**DOI:** 10.3389/fnins.2024.1441799

**Published:** 2024-11-06

**Authors:** Nina M. Ehrhardt, Clara Niehoff, Anna-Christina Oßwald, Daria Antonenko, Guglielmo Lucchese, Robert Fleischmann

**Affiliations:** ^1^Department of Neurology, University Medicine Greifswald, Ferdinand-Sauerbruch-Straße, Greifswald, Germany; ^2^Department of Psychiatry, Psychotherapy and Psychosomatics, Psychiatry University Hospital Zurich, University of Zurich, Lengstrasse, Zurich, Switzerland

**Keywords:** dry EEG, mismatch negativity, theta power, resting-state connectivity, minimum spanning tree, phase lag index

## Abstract

**Background:**

Multipin dry electrodes (dry EEG) provide faster and more convenient application than wet EEG, enabling extensive data collection. This study aims to compare task-related time-frequency representations and resting-state connectivity between wet and dry EEG methods to establish a foundation for using dry EEG in investigations of brain activity in neuropsychiatric disorders.

**Methods:**

In this counterbalanced cross-over study, we acquired wet and dry EEG in 33 healthy participants [*n* = 22 females, mean age (SD) = 24.3 (± 3.4) years] during resting-state and an auditory oddball paradigm. We computed mismatch negativity (MMN), theta power in task EEG, and connectivity measures from resting-state EEG using phase lag index (PLI) and minimum spanning tree (MST). Agreement between wet and dry EEG was assessed using Bland–Altman bias.

**Results:**

MMN was detectable with both systems in time and frequency domains, but dry EEG underestimated MMN mean amplitude, peak latency, and theta power compared to wet EEG. Resting-state connectivity was reliably estimated with dry EEG using MST diameter in all except the very low frequencies (0.5–4 Hz). PLI showed larger differences between wet and dry EEG in all frequencies except theta.

**Conclusion:**

Dry EEG reliably detected MMN and resting-state connectivity despite a lower signal-to-noise ratio. This study provides the methodological basis for using dry EEG in studies investigating the neural processes underlying psychiatric and neurological conditions.

## 1 Introduction

Electroencephalography (EEG) is an important instrument for researching and diagnosing human brain activity. Improved sensor concepts lead to new application areas, particularly for research into the neurophysiological basis and pathophysiological mechanisms of neurological and psychiatric diseases. In this context, EEG can capture brain states that are not observable phenotypically but display disease-specific signal patterns, known as endotypes (Lascano et al., 2016). However, these applications often require multichannel setups beyond the 21 standard electrode positions of routine clinical EEGs, enabling comprehensive signal processing, analysis, and interpretation.

Commercially available multichannel EEG systems with 64 or more channels are based on conventional silver/silver-chloride electrodes in combination with electrolyte gels or pastes (so-called wet EEG). Their application requires well-trained staff for time-consuming and comprehensive preparations, including skin abrasion, gel application, and impedance optimization (Teplan, 2002). This process can cause skin irritation, hair damage (Searle and Kirkup, 2000; Teplan, 2002), and measurement errors due to gel bridges. These drawbacks limit wet EEG’s utility, particularly in large-scale studies and non-laboratory settings, where rapid and repeated measurements are necessary. However, the need for robust and ecologically valid datasets is critical to identify and interpret brain activity patterns associated with specific neuropsychiatric conditions.

The disadvantages of wet EEG led to novel sensor concepts for bioelectrical signal acquisition, including full capacitive sensors, infrared sensors, and dry and quasi-dry contact electrodes (Horng et al., 2012; Liao et al., 2012; Lopez-Gordo et al., 2014). Recently, multipin dry electrodes (dry EEG) that optimally penetrate the hair layer have been applied to multichannel EEG with 64 or more channels (Fiedler et al., 2013; di Fronso et al., 2019). Studies comparing these dry EEG systems to wet EEG have shown that dry EEG offers significant advantages in terms of application speed and participant comfort (di Fronso et al., 2019; Kam et al., 2019; Hinrichs et al., 2020; Fiedler et al., 2022). Additionally, dry EEG produces results similar to wet EEG in resting-state recordings, particularly in higher frequency bands (Fiedler et al., 2015; Hinrichs et al., 2020; Heijs et al., 2021). However, analyses of resting-state power spectra provide only a rough estimate of disease-relevant mechanisms. This study focuses on two key areas to validate dry EEG for measures associated with cognitive functioning and neuropsychiatric disorders: resting-state connectivity and task-related time-frequency representations.

Resting-state connectivity, often analyzed through measures such as phase lag index (PLI; Stam et al., 2007) and graph theory using minimum spanning tree (MST) analysis (Stam et al., 2007; van Diessen et al., 2015), provides insights into functional brain network characteristics (Vecchio et al., 2017). It has been associated with both aging (Otte et al., 2015) and neuropsychiatric disease (Tewarie et al., 2014; van Dellen et al., 2015; Fleischmann et al., 2019). However, it remains to be investigated whether these connectivity measures can be reliably assessed using dry EEG. This assessment is essential for the identification of brain states associated with disease.

In addition to resting-state recordings, task-related activity offers valuable insights into neurophysiological changes associated with neuropsychiatric conditions. Here, event-related potentials (ERPs) - particularly robust EEG components that can be detected in temporal correlation to an experimentally manipulated stimulus (e.g., sound/image sequences)—can help to map brain activity to functional domains of cognition. Some studies have shown that dry EEG can detect certain ERPs, such as visually- and auditory-evoked potentials (VEP/AEP; Fiedler et al., 2015; Heijs et al., 2021; Fiedler et al., 2022) and P300 (Clements et al., 2016; Mathewson et al., 2017; Hinrichs et al., 2020). However, whether task-related activity in the frequency-domain can be reliably assessed using dry EEG remains unclear. Here, an ERP of particular interest is mismatch negativity (MMN). MMN is a frontocentral negative potential elicited 100 to 250 ms after a rarely occurring “deviant” stimulus (“oddball”) surrounded by repeated “standard” stimuli. It is associated with higher cognitive functions, such as attention and memory (Näätänen and Alho, 1995; Näätänen et al., 2007), and neurodegenerative disease (Laptinskaya et al., 2018). In the frequency domain, it has been linked to activity in the theta band (Fuentemilla et al., 2008; Hsiao et al., 2009; Ko et al., 2012), which is often impaired in neuropsychiatric conditions such as schizophrenia (Hua et al., 2023).

Thus, dry EEG is a promising tool for investigations of brain activity for which many aspects of traditional wet EEG have been replicated. However, further validation is necessary to ensure its reliability for connectivity and task-related frequency representation. This study contributes to this effort by comparing wet and dry EEG in detecting MMN in the frequency domain and analyzing resting-state connectivity. This validation is critical for enabling the use of dry EEG in diverse settings, facilitating the collection of extensive clinical datasets.

Therefore, this cross-over study with 33 healthy participants aims to compare wet and dry EEG in detecting MMN, specifically regarding its corresponding theta power. In addition to MMN, we analyze resting-state connectivity using PLI and MST-Diameter across common frequency bands. By validating dry EEG for both time and frequency representations of MMN, as well as for resting-state connectivity, we aim to provide a solid methodological basis for using dry EEG in neurodegenerative or psychiatry applications. We apply Bland–Altman statistics as a measure of agreement between wet and dry EEG which is currently only available for sleep parameters (Leach et al., 2020). The otherwise reported *p*-values only indicate that the alternative hypothesis, in this case, the assumption that EEG measures differ between dry and wet electrodes, cannot be rejected. This does not necessarily indicate that the null hypothesis—the equivalence of the two systems—is true. Similarly, the reported correlations between the systems provide only a measure of relation and not agreement (Altman and Bland, 1983; Giavarina, 2015). As previous validation studies have shown that dry EEG is comparable to wet EEG, we hypothesized that MMN can be detected with dry EEG, i.e., there is a significant difference in amplitude and theta power between standard and deviant tones for both EEG systems. Furthermore, we hypothesized that resting-state connectivity is comparable between wet and dry EEG in alpha and beta frequency but might differ in lower frequencies where significant power differences have been found in previous studies (Fiedler et al., 2015; Hinrichs et al., 2020; Heijs et al., 2021).

## 2 Methods

### 2.1 Participants

Thirty-three participants [*n* = 22 female, mean age (SD) = 24.3 (3.4) years] without underlying psychiatric or neurological disease were recruited through public advertisements. The study was performed in line with the ethical standards outlined in the Declaration of Helsinki and was approved by the local Ethics Committee. All participants provided written informed consent before their participation. Participants were reimbursed with 20 € for their participation.

### 2.2 Oddball paradigm

The auditory oddball paradigm was administered over headphones using E-Prime 2.0 software (Psychology Software Tools, Inc., Pittsburgh, PA, United States) and lasted approximately 20 min. After a few practice trials at the beginning of the session, the sound level was adjusted to a level at which participants reported being able to clearly detect the stimuli. The standard stimulus was a 1,000 Hz tone, while deviant stimuli had frequencies of either 500 Hz or 1,500 Hz. Consecutive stimuli were separated by a stimulus-onset asynchrony (SOA) of 1,555 ms. The two different deviant tones were interspersed among standard tones, occurring 102 times with a 12.5% probability each within a sequence of 612 standard repetitions (75% probability). The presentation order of standard and deviant stimuli was pseudo-randomized, ensuring a minimum of two and a maximum of four standard repetitions between each pair of deviants. Prior to the experimental trials, 10 additional repetitions of the standard stimulus were provided for habituation.

### 2.3 Procedure

All examinations took place on one day in an examination room of the Neurological Outpatient Clinic at University Hospital Greifswald and took approximately two and a half hours. Participants first filled out demographic and anamnestic information on a paper-based form. Then, participants sat in a comfortable chair with armrests and EEG caps were applied. First, the practice trials of the oddball paradigm were performed to determine the individual loudness level. Then, a resting-state recording was performed where participants were instructed to sit quietly, look at a fixation cross on a screen in front of them, and maintain a state of spontaneous flow of thoughts for five min in a relaxed state. For the following oddball paradigm, participants were instructed to ignore the incoming stimuli and focus their attention on a silent movie. Then, the same procedure was completed with the other EEG system. The order of dry and wet EEG was counter-balanced. Participants who completed the procedure with the wet EEG first washed and dried their hair before continuing with the dry EEG.

### 2.4 EEG recording

Wet and dry EEG were recorded with a waveguard touch using eego™ mylab (Advanced Neuro Technologies, Enschede, Netherlands), a sampling rate of 1,024 Hz, and a 512 Hz low-pass filter. The reference and ground electrodes were placed at the left and right mastoid, respectively. Electrode layouts are shown in Supplementary Figure 1.

#### 2.4.1 Wet EEG

The wet EEG was recorded using a 64-channel cap. Three electrodes were placed above, below, and at the outer canthus of the left eye to record the electrooculogram (EOG). The remaining 61 scalp electrodes were mounted according to standard 10–20 electrode positions. The exact electrode layout is depicted in Supplementary Figure 1A. Electrode impedances were kept below 5kΩ.

#### 2.4.2 Dry EEG

The dry EEG was recorded using caps with 64 multipin electrodes. Two electrodes were placed below, and at the outer canthus of the left eye to record the EOG. The remaining 62 scalp electrodes were placed in equidistant positions. The exact electrode layout is depicted in Supplementary Figure 1B. Electrode impedances can significantly exceed 5kΩ, which is an inherent property of the methodology. Therefore, we used the system’s quality index aimed to be “sufficient” (dimension-free measure, arbitrary units) to ensure signal quality.

### 2.5 Data analysis

#### 2.5.1 Preprocessing of EEG data

Preprocessing of the EEG data and the calculation of ERPs were done using MNE Python version 1.4.2 (Gramfort et al., 2013). Data from two participants could not be correctly recorded due to technical failure during recording. First, data was referenced to the average of the mastoids. Then, electrodes containing no signal or substantial artifacts (0–4 per recording for wet EEG, 10–19 per recording for dry EEG) were rejected after visual inspection. Next, data was filtered between 0.1 and 25 Hz using a bandpass filter. Data from the oddball paradigm was epoched into trials starting 0.1 s before stimulus onset until 0.5 s after stimulus onset. Resting-state data was cut into epochs of 8 s. Epoched data was resampled to 512 Hz. For the wet EEG, the signals from EOG electrodes above and below the eye were converted offline to a bipolar vertical EOG signal by re-referencing them against each other. The horizontal EOG was obtained by re-referencing the horizontal EOG next to the left eye against F7. For the dry EEG data, the EOGs below and next to the left eye were re-referenced against 1 L and 1LD for the bipolar vertical and horizontal EOG, respectively. Independent component analysis (ICA) was performed, resulting in 15 components, and components correlating with the EOG signal (threshold = 0.2) were rejected (Jung et al., 2000; Winkler et al., 2011; Hanna et al., 2014; Lucchese et al., 2017). EEG channels with artifacts that had previously been rejected were then interpolated using the spherical spline method. Epochs from the task data with voltage deflections larger than 150 mV peak-to-peak were rejected. Only participants with more than 70% of the trials retained and signal-to-noise ratio (SNR) > 1 in both dry and wet EEG recordings are included in the task analyses (Lopez-Calderon and Luck, 2014; Luck, 2014), resulting in 23 complete datasets for ERP and 32 datasets for resting-state analyses.

#### 2.5.2 Mismatch negativity

ERPs to standard and deviant tones were calculated by averaging over trials and baseline-corrected using the time window from 0.1 before until stimulus onset. The initial 10 standard repetitions and the instances of the standard stimuli occurring immediately after a deviant were excluded from the averages, and the number of trials was equalized for standard and deviant tones and wet and dry EEG for each participant. To calculate MMN, data was extracted from channel FCz (wet EEG) and 3Z (dry EEG) in the time window of 100 to 150 ms after stimulus onset and amplitude in response to standard stimuli was subtracted from deviant tones. Peak latency and amplitude were determined with the MNE *get_peak*-function, which determines the maximum amplitude and its time point in the given time window and channel.

##### 2.5.2.1 Theta power

Time-frequency analysis was conducted with the Fieldtrip toolbox (Oostenveld et al., 2011) in Matlab (The MathWorks, Natick, MA) using a Morlet wavelet with a varying number of cycles increasing from 3 to 10 (wavelet length m = 2; normalization factor A = σt − 1/2 *π* − 1/4) in a frequency range from 1 to 18 Hz in frequency bins of 1 Hz and time window 1.2 s before and after stimulus onset, respectively (a total of 2.4 s), in time bins of 0.05 s (Roach and Mathalon, 2008; Garagnani et al., 2016). Trials for standard and deviant tones were averaged for each participant and EEG system separately, and subsequently, decibel (dB) baseline-corrected in the time window 600 to 300 ms before stimulus onset. Then, theta (4–8 Hz) power 100 to 300 ms after stimulus onset was extracted from channel FCz (wet EEG) and 3Z (dry EEG) for subsequent statistical analyses.

##### 2.5.2.2 Signal-to-noise ratio

The SNR is calculated through dividing the signal by an estimate of noise. Traditionally, the signal is defined as the power at peak latency, while noise is defined by the mean power in a baseline period before the signal of interest. To estimate the noise, we used the plus-minus procedure by Schimmel (1967) instead because it allows signal and noise to be estimated in the same time window, leading to a more conservative estimation of SNR (Viola et al., 2011). First, the signal was calculated for each participant by averaging all deviant trials from channel FCz (wet EEG) or 3Z (dry EEG) in the time window of 100 to 150 ms. Then, the noise was estimated by flipping the polarity of every other deviant trial from channel FCz (wet EEG) or 3Z (dry EEG) in the time window of 100 to 150 ms before averaging over trials (Schimmel, 1967; Viola et al., 2011). This procedure leads all time-locked features to sum to zero while the noise remains in the average. Finally, the SNR was calculated by dividing the root mean square (RMS) of the signal by the RMS of the estimated noise (Hanna and Pulvermüller, 2014; Lucchese et al., 2017).

#### 2.5.3 Resting-state connectivity

Connectivity analyses were performed in Brainwave (*Version 0.9.165.57*; van Dellen et al., 2018). Data was referenced to an average reference, bandpass filtered to extract activity in common frequency bands - delta (0.5-4 Hz), theta (4-8 Hz), alpha (8-13 Hz) and only lower beta frequencies (13-20 Hz) to mitigate the impact of muscle activity (Whitham et al., 2007) on connectivity measures. PLI was used to calculate functional connectivity strength (Stam et al., 2007). The PLI is based on Hilbert-transformed instantaneous phase differences, capturing the asymmetry in phase leading and lagging between two signals. Its values range between 0 and 1; 1 indicates complete phase locking, and values reaching 0 mean no phase synchronization or equal in leading and lagging over the epoch. An average over all PLI values between all channel pairs was calculated per epoch and averaged to a mean PLI per frequency band per subject. MST was derived from PLI. Since MST algorithms work on distances, PLI values were transformed into distance measures. The resulting MST is a subset of the graph that connects all nodes (channels) with the minimum possible total edge weight (distance) and without any cycles. The diameter of an MST (D) is the longest path between any two nodes in the MST (d) defined by the number of steps required to get from one node to another node (the number of links between two nodes; Stam and van Straaten, 2012). It is corrected for the total number of links (M): D = d/M (Tewarie et al., 2014). MST diameter reflects the greatest distance over which information or synchronization is transmitted within the network (with greater values indicating a less efficient network; Numan et al., 2017).

#### 2.5.4 Statistical analyses

To determine whether brain activity in the oddball paradigm can be distinguished with both wet and dry EEG, two repeated-measures ANOVA with the factors EEG system (wet versus dry) and tone (standard versus deviant) were applied with the mean amplitude and mean theta power of the specified channel and time window as the dependent variable. In case of a significant interaction, follow-up one-sided dependent *t*-tests were conducted with Bonferroni correction to correct for multiple testing. An alpha level of 0.05 was applied for all tests. Additionally, peak latency and amplitude of MMN were compared between wet and dry EEG with two-sided dependent *t*-tests.

Additionally, Bland–Altman statistics (Altman and Bland, 1983; Giavarina, 2015) were conducted to test the agreement between wet and dry EEG for ERP characteristics (MMN mean and peak amplitude, peak latency; theta power; PLI and MST-Diameter in different frequency bands) using *blandr* (Datta, 2017). Here, the bias of dry EEG was calculated compared to wet EEG as the “gold standard” by taking the mean difference between wet and dry EEG of the corresponding value. A mean difference of zero would indicate complete agreement between measurement methods. We report 95% confidence intervals of the bias to determine statistical significance. Lower and upper limits of agreement were determined as values falling within 95% around the mean difference. Bland Altman plots show the difference between wet and dry EEG in the corresponding measure plotted against the mean between wet and dry EEG as approximation of the true value (Altman and Bland, 1983).

## 3 Results

### 3.1 Data quality

**Figure 1:**
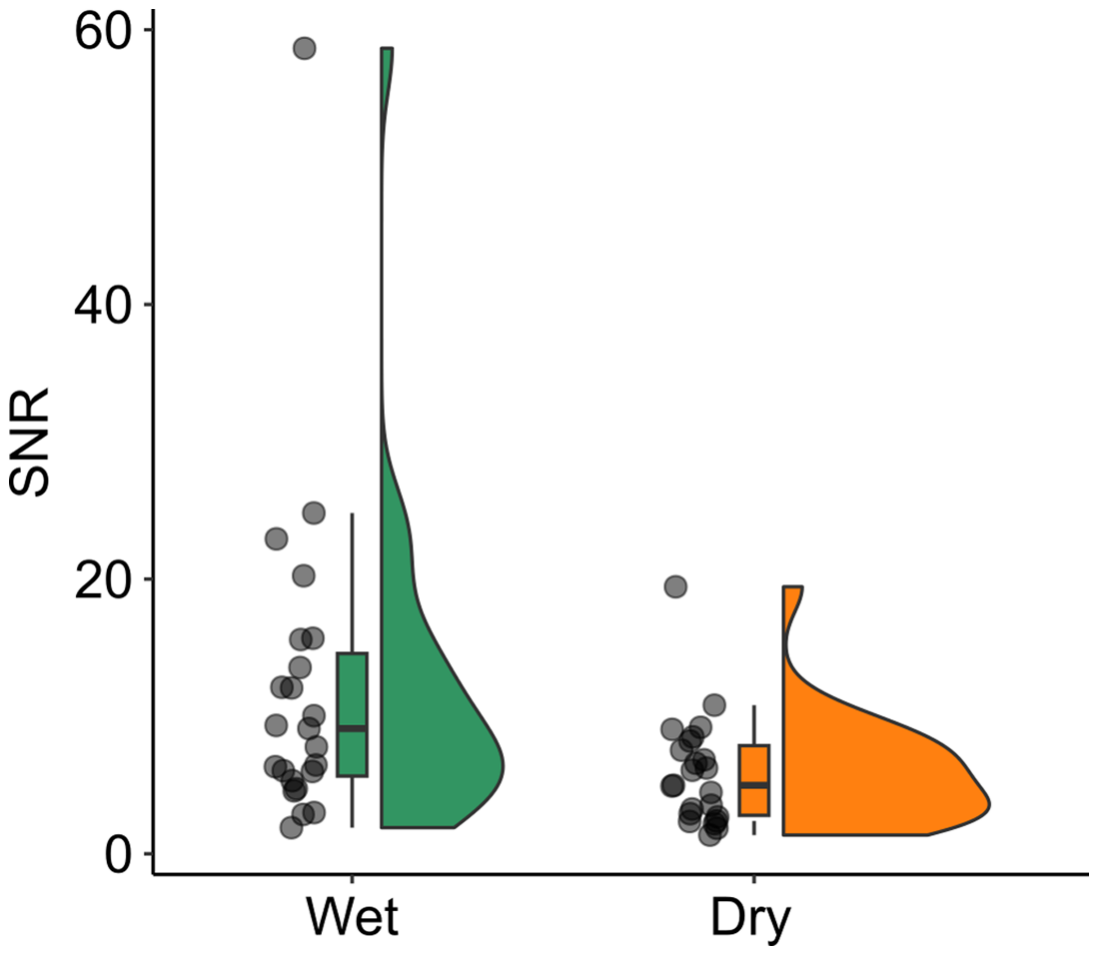
SNR for wet and dry EEG. The SNR is shown with scattered dots indicating individual participants, and boxplots showing the median, first and third quartile, and 1.5 interquartile range. SNR, signal-to-noise ratio.

Rejected channels, components and trials, and SNR of MMN were compared between EEG systems using a dependent *t*-test (see Supplementary Table 1 for detailed results). For dry EEG, significantly more channels and trials were rejected than for wet EEG (*t*(22)s < −9.22, *p*s < 0.001). The SNR of MMN was significantly lower for dry than for wet EEG (*t*(22) = 2.26, *p* = 0.034; see Figure 1).

### 3.2 Mismatch negativity

**Figure 2:**
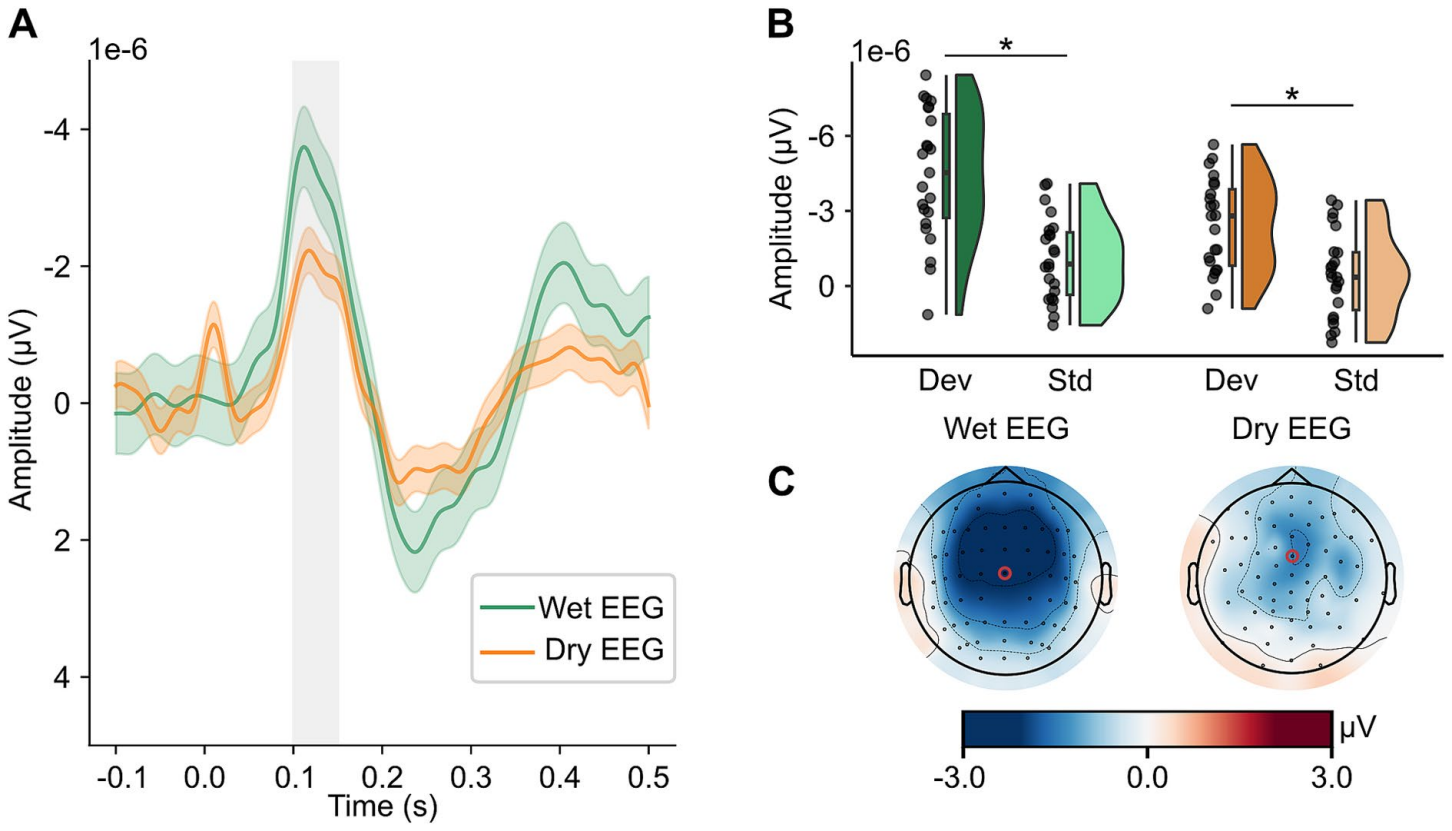
MMN can be detected in wet and dry EEG. In panel A, MMN amplitude (deviant – standard tones) in channels FCz (wet EEG) and 3Z (dry EEG) is depicted for wet and dry EEG with a 95% confidence interval. The time window used to determine mean amplitude and used for statistical analyses is indicated by the shaded gray area. In panel B, mean amplitude in response to deviant and standard tones is shown for wet and dry EEG with their distribution displayed by density plots. Scattered dots indicate individual participants, boxplots show the median, first and third quartile, and 1.5 interquartile range. In panel C, topographies of MMN averaged in the time window from 100 to 150 ms are shown for both wet (left) and dry (right) EEG with a red circle marking the channels used for analyses. All data is baseline-corrected with a baseline time window from 100 ms before until stimulus onset. μV, microvolts. ms, milliseconds. Std, standard tone. Dev, deviant tone. MMN, mismatch negativity.

Figure 2 shows the ERP curves over the whole epoch (0.1 s before stimulus onset until 0.5 s after stimulus onset) with the time window used for analysis (100 to 150 ms after stimulus onset) indicated by the shaded gray area (Figure 2A), mean amplitude responses to standard and deviant tones (Figure 2B), and topographical maps of MMN (Figure 2C) for both EEG systems. There was a significant EEG system (wet vs. dry) x tone (standard vs. deviant) interaction on mean MMN amplitude (*F*(22) = 13.276, *p* = 0.001, *η_p_^2^* = 0.38). Post-hoc dependent *t*-tests indicated that with both EEG systems, the amplitude following deviant tones was significantly more negative than following the standard tones (see Figure 2B; wet EEG: *t*(22) = −11.20, *p*_corr_ < 0.001, *d* = 2.33; dry EEG: *t*(22) = −5.17, *p*_corr_ < 0.001, *d* = 1.08), indicating that MMN could be detected with both EEG systems. However, the mean difference between standard and deviant tones differed between wet (mean (SD) = −3.35e-06 (1.44e-06) μV) and dry (mean (SD) = −1.96e-06 (1.82e-06) μV) EEG (*t*(22) = −3.64, *p* = 0.001, *d* = 0.76), thus explaining the significant interaction term between EEG system and tone. The peak amplitude of MMN did not differ significantly between wet (mean (SD) = −2.70e-7 (1.72e-6) μV) and dry (mean (SD) = −3.55e-7 (2.89e-6) μV) EEG (*t*(22) = 0.11, *p* = 0.911, *d* = 0.02) EEG. However, the peak amplitude was reached significantly earlier when measured with dry (mean (SD) = 126 (15) ms) compared to wet (mean (SD) = 138 (13) ms) EEG (*t*(22) = 2.52, *p* = 0.02, *d* = 0.53) EEG. The results for other fronto-central electrodes were overall similar to FCz/3Z (see Supplementary Table 2).

**Figure 3:**
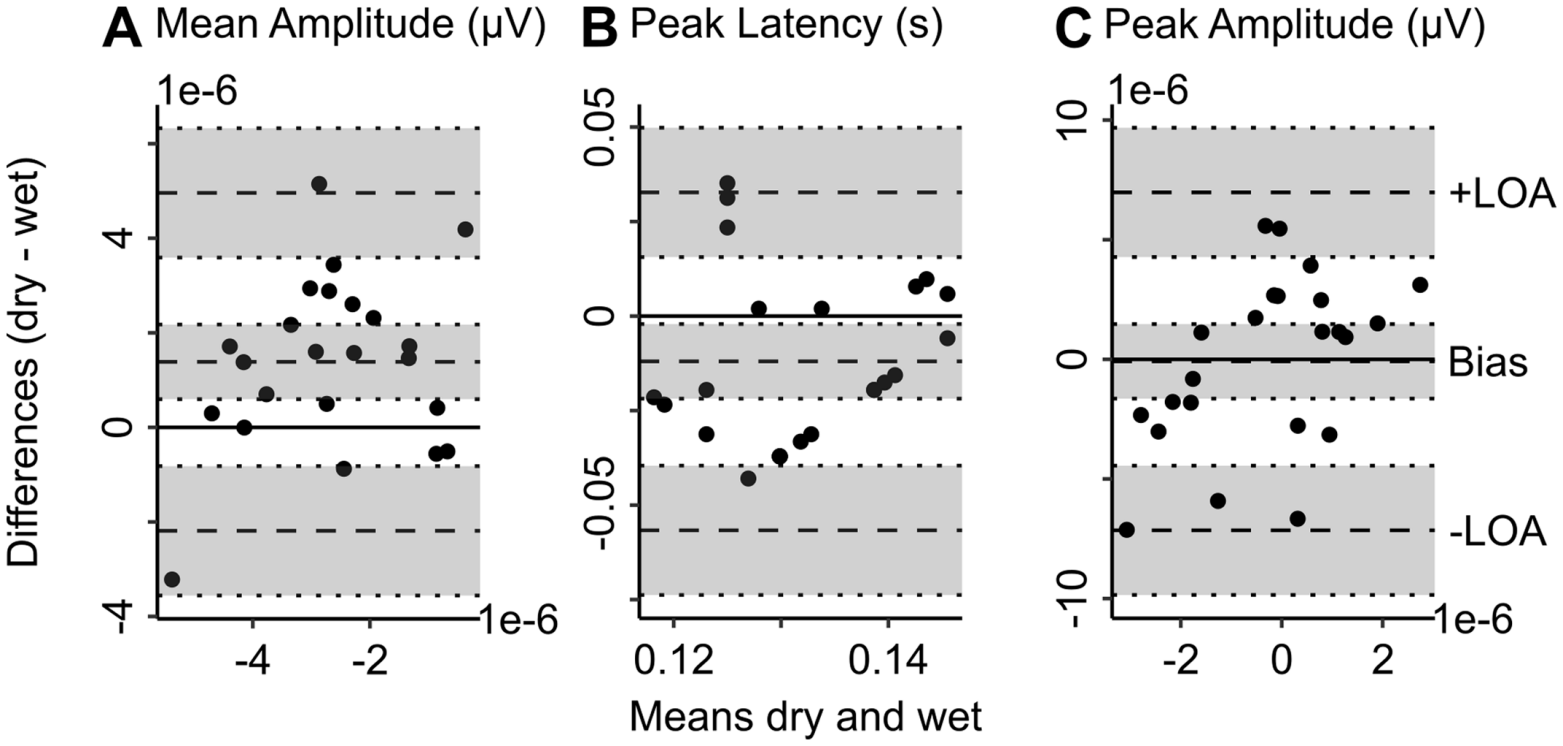
Bland Altman plots show the level of agreement between wet and dry EEG for MMN characteristics. Bland Altman plots show the difference between wet and dry EEG (y-axis) in the corresponding measure plotted against the mean between wet and dry EEG (x-axis) of the corresponding value as approximation of the true value (Altman and Bland, 1983) for each participant. Panel A shows the difference in baseline-corrected (−100 to 0 ms) MMN averaged at channel FCz (wet EEG) and 3Z (dry EEG) 100 to 150 ms after stimulus onset between wet and dry EEG for each participant plotted against the mean MMN in wet and dry EEG as approximation of the true MMN amplitude values. Panel B shows the difference in peak latency of MMN between wet and dry EEG plotted against the mean peak latency of the both systems. Panel C shows the difference in peak amplitude between wet and dry EEG plotted against the mean peak amplitude of the two EEG systems. The scatterplots show individual participants. Dotted lines represent the bias and lower (−) and upper (+) limit of agreement as indicated, with 95% confidence intervals indicated by shaded gray areas. The lower and upper limits of agreement are determined as the values falling within 95% around the mean difference. MMN, mismatch negativity. –LOA, lower limit of agreement. +LOA, upper limit of agreement.

The Bland–Altman bias for mean amplitude (bias = 1.38e-6 μV, 95% CI = [5.97e-7, −2.17e-6]) shows that the difference scores between deviant and standard tones tend to be underestimated with dry compared to wet EEG (see Figure 3A). The peak latency is also slightly underestimated with dry compared to wet EEG (see Figure 3B; bias = −0.01 s, 95% CI = [−0.002, −0.02]). The confidence interval for the bias of the peak amplitude contains zero, indicating that the peak amplitude is comparable between wet and dry EEG (see Figure 3C; bias = −8.50e-8 μV, 95% CI = [−1.64e-6, 1.47e-6]). Additionally, for all MMN characteristics, the individual values fall within the upper and lower limits of agreement.

#### 3.2.1 Theta power

**Figure 4:**
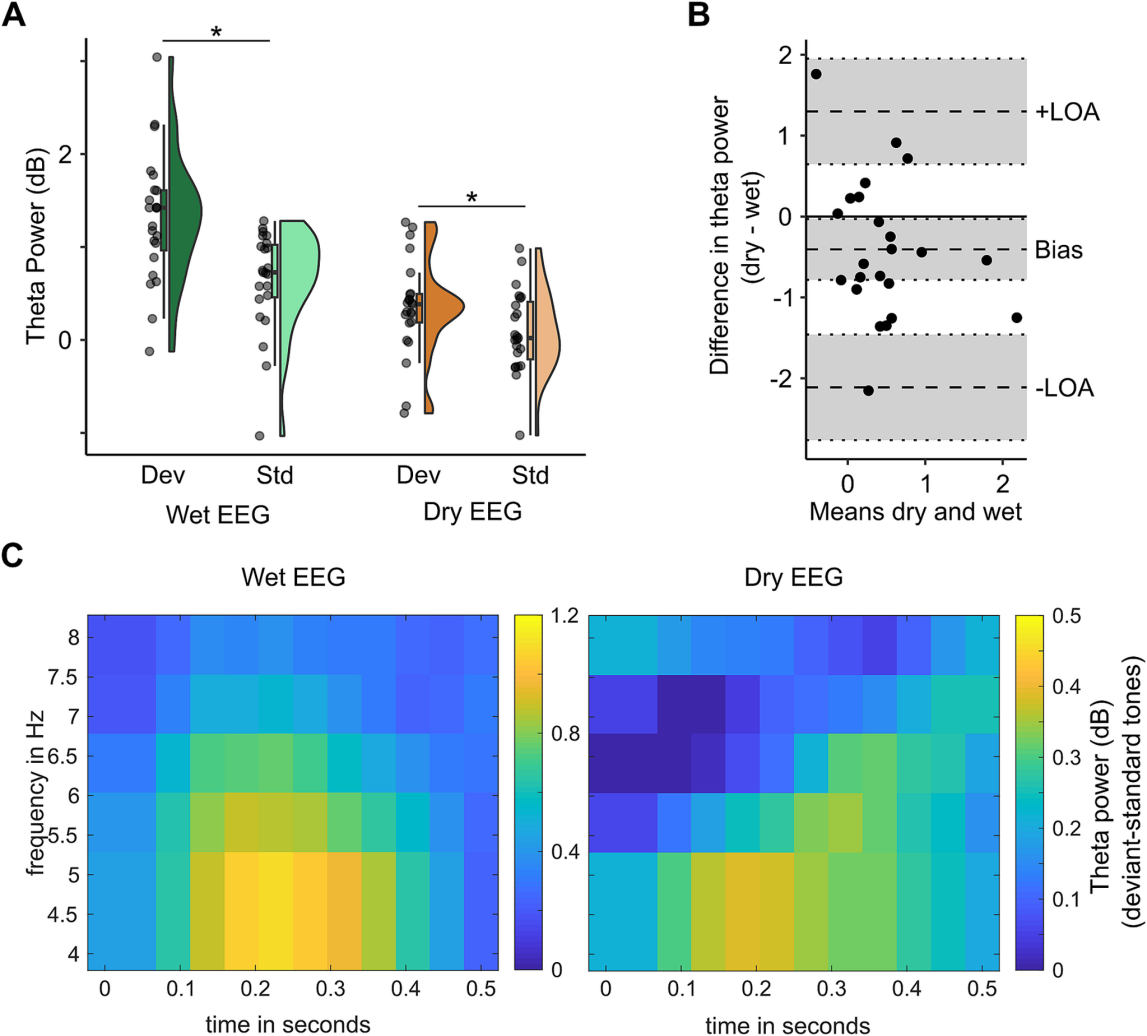
Theta power in the oddball paradigm can be distinguished with both wet and dry EEG. In panel A, mean dB baseline-corrected theta power in response to deviant and standard tones is shown for channels FCz (wet EEG) and 3Z (dry EEG) with their distribution displayed by density plots. Scattered dots indicate individual participants, boxplots show the median, first and third quartile, and 1.5 interquartile range. Panel B shows the difference in theta power between wet (channel FCz) and dry (channel 3Z) EEG (y-axis) for each participant plotted against the mean of theta power in wet and dry EEG (x-axis) as approximation of the true theta power values. Panel C shows the power spectra for wet (left) and dry (right) EEG at channel FCz (wet) and 3Z (dry) EEG.

For theta power, there was a significant EEG system (wet vs. dry) x tone (standard vs. deviant) interaction (*F*(22) = 5.014, *p* = 0.036, *η_p_^2^* = 0.19). Post-hoc dependent *t*-tests indicated that theta power was higher following deviant compared to standard tones for both EEG systems (see Figure 4A; wet EEG: *t*(22) = 3.98, *p*_corr_ < 0.001, *d* = 0.83; dry EEG: *t*(22) = 2.09, *p*_corr_ = 0.048, *d* = 0.44), indicating that theta associated with MMN could be detected with both EEG systems. However, the mean difference between standard and deviant tones differed between wet (mean (SD) = 0.67 (0.81) dB) and dry (mean (SD) = 0.27 (0.61) dB) EEG (*t*(22) = −2.24, *p* = 0.036, *d* = 0.47), thus explaining the significant interaction term between EEG system and tone (see Figure 4C). The Bland–Altman bias for theta power (bias = −0.41 dB, 95% CI = [−0.78, −0.03]) shows that the difference in theta power between deviant and standard tones is slightly underestimated with dry compared to wet EEG (see Figure 4B).

### 3.3 Sensor-level resting-state connectivity

**Figure 5:**
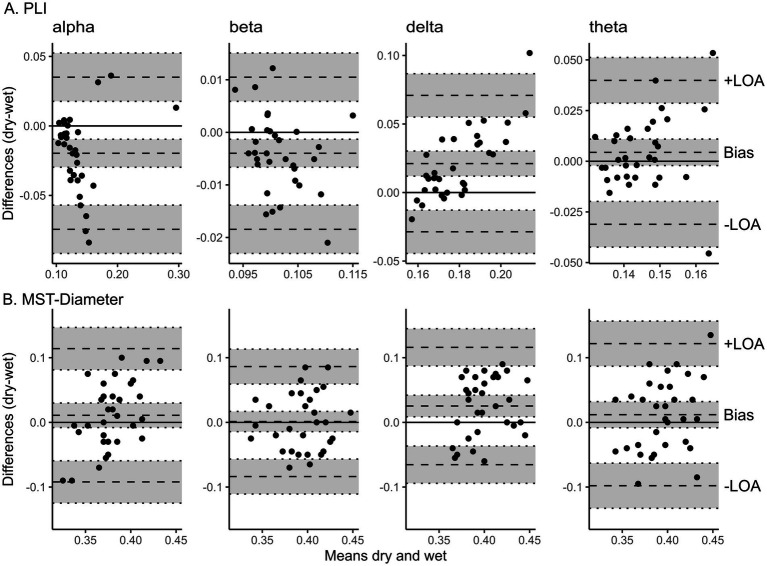
Bland Altman plots show the level of agreement between wet and dry EEG for resting-state connectivity. Bland Altman plots show the difference between wet and dry EEG (y-axis) in the corresponding measure plotted against the mean between wet and dry EEG (x-axis) of the corresponding value as approximation of the true value (Altman and Bland, 1983) for each participant. Panel A shows the difference in PLI averaged across all electrodes between wet and dry EEG for each participant plotted against the mean PLI in wet and dry EEG (x-axis) as approximation of the true value for alpha (8-13 Hz), beta (13-20 Hz), delta (0.5-4 Hz), and theta (4-8 Hz) frequency (from left to right). Panel B shows the difference in MST-Diameter derived from PLI between all channel pairs between wet and dry EEG plotted against the mean MST-Diameter of the both systems for alpha (8-13 Hz), beta (13-20 Hz), delta (0.5-4 Hz), and theta (4-8 Hz) frequency (from left to right). The scatterplots show individual participants. Dotted lines represent the bias and lower (−) and upper (+) limit of agreement as indicated, with 95% confidence intervals indicated by shaded gray areas. The lower and upper limits of agreement are determined as the values falling within 95% around the mean difference. PLI, phase lag index. MST, minimum spanning tree. –LOA, lower limit of agreement. +LOA, upper limit of agreement.

The Bland–Altman bias is reported for PLI and MST-Diameter with a 95% confidence interval separately for each frequency band in Table 1. The Bland–Altman plots are shown in Figure 5 and the distribution of raw values is depicted in Supplementary Figure 2. PLI was significantly underestimated for alpha and beta frequency and significantly overestimated for delta frequency (see Figure 5A). In theta frequency, the PLI measured with dry EEG was slightly higher, but overall, it was similar to wet EEG (95% confidence interval of bias includes zero, see Figure 5A, right). Overall, PLI was within the limits of agreement for most values except three extreme values for lower frequency bands (see Figure 5A). The MST-Diameter could be more reliably measured with dry EEG than PLI. Here, the 95% confidence interval of the bias includes zero for alpha, beta, and theta connectivity (see Figure 5B), indicating that the MST-Diameter is very similar measured with dry and wet EEG in these frequency bands. Only in delta frequency is MST-Diameter significantly overestimated with dry compared to wet EEG (see Figure 5B). However, for MST-Diameter, all values fall within the limits of agreement (see Figure 5B). The results acquired using Bland–Altman statistics are overall comparable with frequentist comparisons between wet and dry EEG using Wilcoxon *t*-tests to correct for non-normal distribution of the data, which we report in Supplementary Table 3 for power and multiple additional connectivity measures. Here, the effect sizes show that the differences in PLI range from small (theta) to large (delta), while the differences in MST-Diameter are small or even negligible except for delta frequency (Cohen, 1992; see Table 1).

**Table 1:** Bland–Altman bias with 95% confidence interval and effect size for wet vs. dry comparison for resting-state connectivity.

Frequenz	Bias (95% CI)	*d*
Phase-lag index		
Delta (0.5-4 Hz)	2.11 e-2 (1.12e-2; 3.03e-2)	−0.55 (moderate)
Theta (4-8 Hz)	0.44e-2 (−0.21e-2; 1.09e-2)	0.20 (small)
Alpha (8-13 Hz)	-1.97e-2 (−2.97e-2; −0.96e-2)	0.70 (moderate)
Beta (13–20 Hz)	−0.40e-2 (−0.66e-2; −0.13e-2)	0.54 (moderate)
MST-Diameter		
Delta (0.5-4 Hz)	2.53e-2 (0.86e-2; 4.20e-2)	−0.83 (large)
Theta (4-8 Hz)	1.19e-2 (−0.83e-2; 3.21e-2)	−0.21 (small)
Alpha (8-13 Hz)	1.09e-2 (−0.80e-2; 2.99e-2)	−0.21 (small)
Beta (13–20 Hz)	0.13e-2 (−1.44e-2; 1.69e-2)	−0.03 (negligible)

MST, minimum spanning tree.

## 4 Discussion

Developing, improving, and validating methods to investigate the neural processes underlying cognition is crucial for diagnosing and treating psychiatric and neurological conditions. This counterbalanced cross-over study contributes to this effort by comparing wet and dry EEG in measuring connectivity and task-related frequency representation. In line with our hypothesis, we show that the neural response to infrequently occurring deviant auditory stimuli can be distinguished from frequently occurring standard tones using multipin dry EEG in both the time and frequency domain. These results indicate that cognitive ERPs, commonly investigated using the standard wet EEG, such as MMN, can also be reliably detected using dry EEG. Furthermore, we show that resting-state connectivity can be assessed with dry EEG with results similar to wet EEG. Here, MST-Diameter was more in line with values from wet EEG than PLI.

Building on previous research demonstrating the comparability of wet and dry EEG for resting-state power and ERPs (Fiedler et al., 2015; Clements et al., 2016; Mathewson et al., 2017; Kam et al., 2019; Hinrichs et al., 2020; Heijs et al., 2021; Fiedler et al., 2022), our study further investigates this comparability by measuring the agreement between high-density wet and dry EEG for resting-state connectivity and MMN in time and frequency domains. While P300 as a cognitive ERP and AEP as an auditory ERP could be detected with both wet and dry EEG (Clements et al., 2016; Mathewson et al., 2017; Kam et al., 2019; Hinrichs et al., 2020; Heijs et al., 2021), our results extend these findings for MMN and by assessing the degree of agreement between the two methods regarding ERP characteristics. Previous studies found that P300 amplitudes are highly correlated (Kam et al., 2019), and amplitude and peak latencies do not differ significantly between systems for P300 or AEP (Hinrichs et al., 2020; Heijs et al., 2021). Contrary to this and our initial expectations, however, statistics assessing the agreement between the two methods show that key characteristics of MMN, such as peak latency and peak and mean amplitude, can differ between wet and dry EEG. This finding aligns with a study using dry EEG for a brain-computer interface, which also found significantly different P300 amplitudes between wet and dry EEG (Clements et al., 2016). As ERP peak measures are easily influenced by noise (Luck, 2014), this could have resulted in our study’s significant differences in MMN mean and peak amplitude, and latency. Therefore, while detecting ERPs of higher cognitive functions using dry EEG is possible, the specific measures should not be compared with previous wet EEG results but rather with a control group assessed with dry EEG. In addition to the time domain of MMN, we extend previous studies by showing that condition differences in the frequency domain can also be detected with dry EEG. Specifically, there was a significant difference in theta power between standard and deviant tones for wet and dry EEG. Although this difference was smaller for dry compared to wet EEG, our results show that with appropriate task design, including sufficiently long baseline windows and a large number of trials to increase SNR, dry EEG provides a promising tool to investigate pathologically relevant changes in both time and frequency domain, not only in resting-state but also in response to cognitive stimuli.

In resting state, we extend previous studies by assessing brain network connectivity. Notably, differences between wet and dry EEG emerge in lower frequency bands, such as delta (0.5–4 Hz) and theta (4–8 Hz). This finding aligns with studies showing significant differences in resting-state power in these frequencies due to artifacts in dry EEG data (Hinrichs et al., 2020; Heijs et al., 2021). In our study, MST-Diameter showed more reliable results in dry EEG compared to wet EEG than PLI, although MST parameters are derived from PLI. A plausible explanation for this discrepancy is that absolute measures of PLI might differ, i.e., the amount of phase coupling might differ to some extent for slow frequencies, yet their order in a network remains rather unaffected, rendering the MST structure consistent across methods. Despite these differences, most variations between wet and dry EEG fall within the limits of agreement. Furthermore, effect sizes for the difference between wet and dry EEG range from negligible to moderate for all frequencies except delta.

### 4.1 Limitations and future directions

While our findings extend the comparability of dry EEG with wet EEG, whether the SNR of dry EEG is sufficient to detect more fine-grained condition and/or group differences, especially for connectivity measures, remains to be investigated. Lower SNR in dry compared to wet EEG is a common issue (Mathewson et al., 2017; Hinrichs et al., 2020; Heijs et al., 2021), even though previous studies have already rejected more trials (Hinrichs et al., 2020; Heijs et al., 2021). The lower SNR in dry compared to wet EEG could result from reduced skin-to-electrode contact (Clements et al., 2016), making it more susceptible to movement artifacts (di Fronso et al., 2019). Indeed, our data contained many jump artifacts resulting from sudden impedance changes likely caused by low skin-to-electrode contact. Yet, other studies have found comparable signal quality between wet and dry EEG. However, significantly more channels were rejected in these studies, possibly providing better signal quality in the remaining data (Kam et al., 2019; Fiedler et al., 2022). Despite the potentially reduced signal quality, dry EEG offers two significant advantages, especially in clinical settings: faster application (Fiedler et al., 2015; di Fronso et al., 2019; Hinrichs et al., 2020; Heijs et al., 2021; Fiedler et al., 2022) and greater comfort for the participants (Hinrichs et al., 2020; Fiedler et al., 2022). Additionally, dry EEG has been proposed for use in home settings (e.g., Kam et al., 2019; Hinrichs et al., 2020), appealing for longer-term clinical or research monitoring. Future research can build on our findings to investigate cognitive ERPs eliciting robust experimental condition or group differences with dry EEG in ecologically valid settings and vulnerable groups where high-density wet EEG might not be feasible, such as delirious patients. Thus, the faster, more convenient application of dry EEG could enhance the understanding of the mechanisms underlying various psychiatric and neurological conditions, providing crucial insights for developing more effective interventions or treatments.

## 5 Conclusion

In sum, our study showed that MMN can be detected using dry EEG in time- and frequency domains, although ERP characteristics might differ when assessed with dry compared to wet EEG. Additionally, we showed that assessing resting-state connectivity with high-density dry EEG is a promising tool for investigating disease-related changes in brain networks, but the reliability of the results depended on the specific measure and frequency used. Nevertheless, resting-state connectivity and time- and frequency representations of cognitive ERPs obtained with dry EEG could be used in future studies to advance understanding of the neurophysiological mechanisms of psychiatric and neurological conditions where high-density wet EEG is not possible.

## Data Availability

Unrestricted sharing of data generated in this study is prohibited by the General Data Protection Regulation. The datasets are, however, available from the corresponding author upon reasonable request. Code used for EEG-preprocessing, ERP, and statistical analyses can be found here: https://github.com/nina174/FrontNeurosc_TroNa.
