# Supplementary figures and images for: Comparison of dry and wet electroencephalography for the assessment of cognitive evoked potentials and sensor-level connectivity
Source: Front Neurosci. 2024 Nov 6;18:1441799. doi: 10.3389/fnins.2024.1441799 (PMC11576458; doi:10.3389/fnins.2024.1441799)

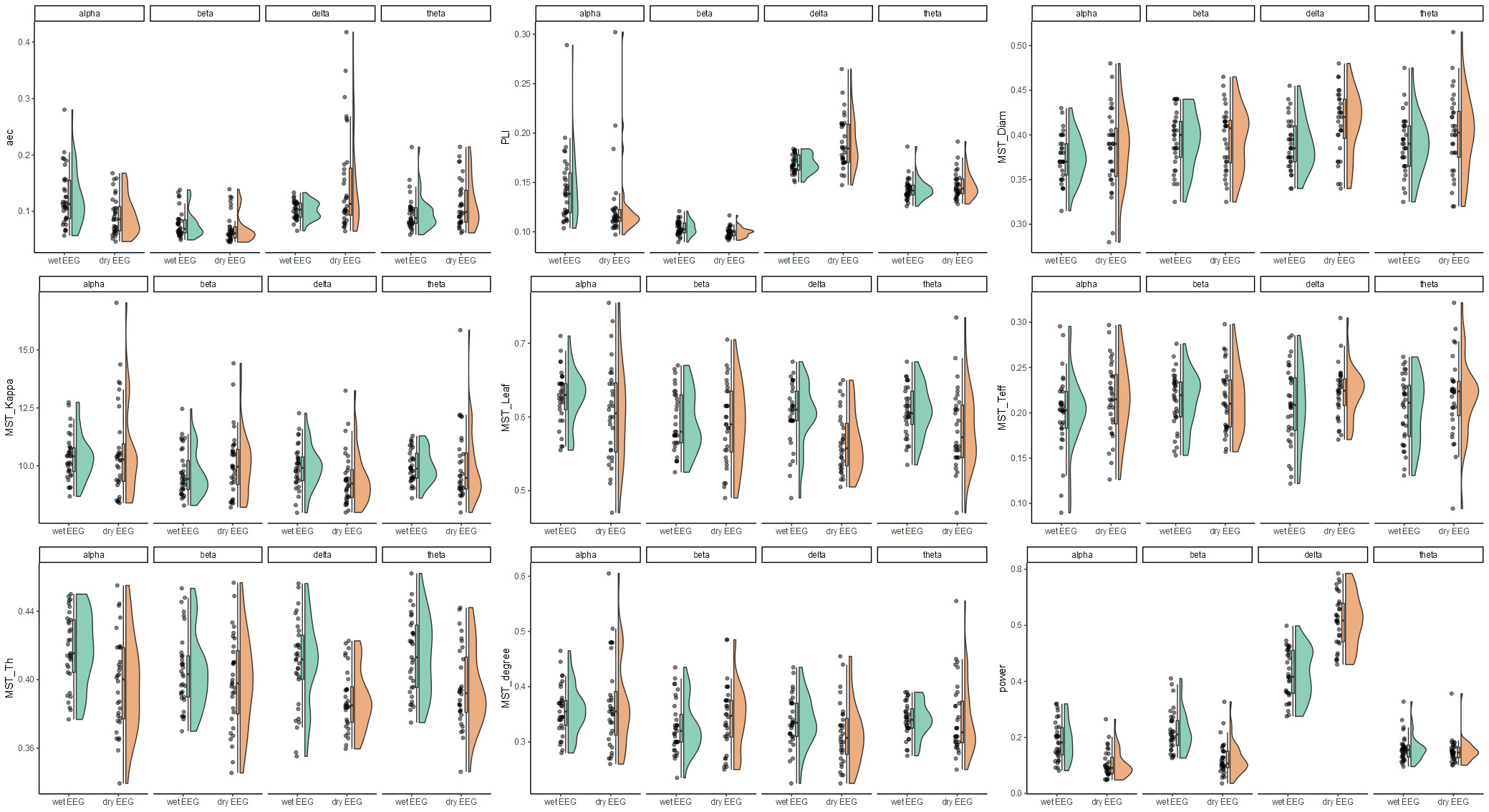

Supplement: Supplementary file 1 [file Image_1.jpeg]
