# Supplementary material for: Comparison of dry and wet electroencephalography for the assessment of cognitive evoked potentials and sensor-level connectivity
Source: Front Neurosci. 2024 Nov 6;18:1441799. doi: 10.3389/fnins.2024.1441799 (PMC11576458; doi:10.3389/fnins.2024.1441799)
Supplement: Supplementary file 2 [file Table_1.pdf]

# *Supplementary Material*

**Supplementary Table 1.**

*Overview of rejected channels, components, and trials during preprocessing, and SNR for participants included in the analyses (task data: n=23, resting-state data: n = 32)*

| | Wet EEG | | | Dry EEG | | | $t$(df), $p_{uncorr}$ |
|---|---|---|---|---|---|---|---|
| | Minimum | Maximum | Mean (SD) | Minimum | Maximum | Mean (SD) | |
| Rejected channel | 0 | 4 | 0.48 (0.95) | 10 | 19 | 15.39 (2.19) | $t(22) = -29.44, p < .001$ |
| Rejected components | | | | | | | |
| Task | 1 | 4 | 2.35 (0.71) | 1 | 6 | 2.35 (1.30) | $t(22) < 0.01, p > .99$ |
| Resting-state | 1 | 7 | 3.06 (1.61) | 0 | 5 | 2.62 (1.24) | $t(31) = 1.16, p = .256$ |
| Rejected trials task (%) | 0 | 3.03 | 0.54 (0.82) | 2.54 | 24.58 | 13.86 (7.12) | $t(22) = -9.22, p < .001$ |
| SNR | 1.90 | 58.65 | 12.14 (11.96) | 1.36 | 19.44 | 5.89 (4.02) | $t(22) = 2.26, p = .034$ |

*Note.* SD = standard deviation. SNR = Signal-to-Noise Ratio.

## Supplementary Methods

Power spectral analysis was performed using a Fast Fourier Transform (FFT). Power spectra were calculated for delta (0.5-4Hz), theta (4-8Hz), alpha (8-13Hz) and beta (13-30Hz) frequency bands. The amplitude envelope correlation was used as a measure of amplitude coupling between signals. It measures the Pearson correlation between the envelopes of the amplitudes of the signals, which are obtained by using the Hilbert transform of the time series (Bruns et al., 2000). Here, we used the version corrected for spatial leakage or volume conduction (AECc) (Hipp et al., 2012). The correction for volume conduction is achieved by pairwise orthogonalization between signals X and Y (in both directions X to Y and Y to X) before calculation of the Pearson correlation coefficient. The positive part of the correlation coefficient was used to estimate connectivity strength, which ranges between 0-1.

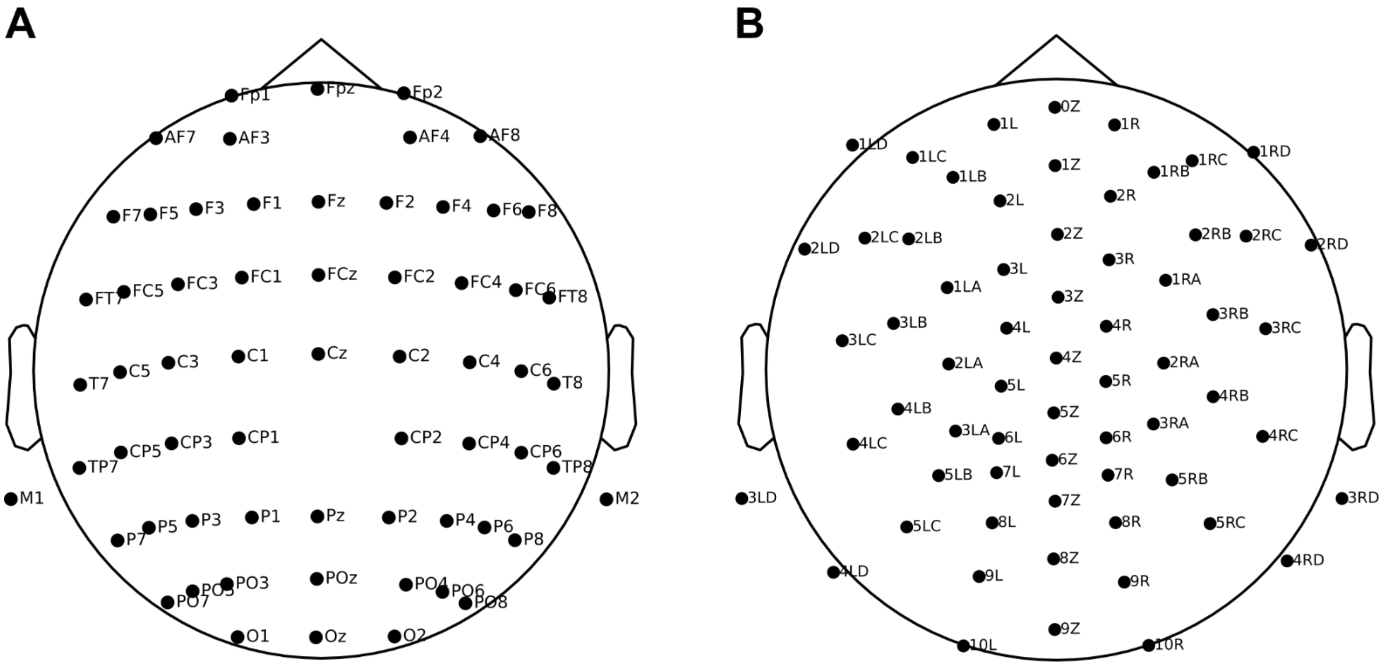


**Supplementary Figure 1. Electrode layouts.** Panel A shows the electrode placement for wet EEG. Panel B shows the electrode placement for dry EEG.

**Supplementary Table 2.**

*Comparison of MMN between wet and dry EEG in other fronto-central electrodes*

| Interaction | Mean amplitude | | | | | Peak amplitude | | | | Peak latency | | | |
|---|---|---|---|---|---|---|---|---|---|---|---|---|---|
| | Wet EEG | | Dry EEG | | | | | | | | | | |
| | Mean (SD) | Deviant vs. standard tones | Mean (SD) | Deviant vs. standard tones | BA bias (95% CI) | Mean (SD) wet EEG | Mean (SD) dry EEG | Wet vs. dry EEG | BA bias (95% CI) | Mean (SD) wet | Mean (SD) dry | Wet vs. dry EEG | BA bias (95% CI) |
| **Cz/4Z** | | | | | | | | | | | | | |
| $F(22) = 12.015$<br>$p = .002$<br>$\eta_p^2 = 0.35$ | -3.24e-06 (1.39e-06) | $t(22) = -11.2$<br>$p < .001$<br>$d = -2.33$ | -1.87e-06 (1.97e-06) | $t(22) = -4.55$<br>$p < .001$<br>$d = -0.95$ | 1.38e-06 (5.54e-07 to 2.20e-06) | 0.47e-06 (1.57e-06) | 1.13e-6 (3.08e-06) | $t(22) = 0.93$<br>$p = 0.363$<br>$d = 0.19$ | -6.64e-07 ( -2.15e-06 to 8.18e-07) | 0.13 (0.02) | 0.122 (0.02) | $t(22) = 2.28$<br>$p = .032$<br>$d = 0.48$ | -0.01 ( -0.02 to 0.00) |
| **Fz/2Z** | | | | | | | | | | | | | |
| $F(22) = 10.347$<br>$p = .004$ | -2.86e-06 (2.63e-06) | $t(22) = -10.8$<br>$p < .001$ | -1.83e-06 (1.83e-06) | $t(22) = -6.77$<br>$p < .001$ | 1.24e-06 (4.41e-07 to | 0.08e-06 (1.66 e-06) | 0.19e-06 (1.97e-06) | $t(22) = 0.18$ | -1.09e-07 ( -1.36e-06 to | 0.13 (0.02) | 0.12 (0.02) | $t(22) = 0.30$ | < 0.01 ( -0.01 to 0.01) |

| | | | | | | | | | | | | | |
|---|---|---|---|---|---|---|---|---|---|---|---|---|---|
| $\eta_p^2$ = 0.32 | | *d* = -2.26 | | *d* = -1.41 | 2.04e-06) | | | *p* = 0.857<br>*d* = 0.04 | 1.14e-06) | | | *p* = 0.77<br>*d* = 0.06 | |
| | | | | | | **FC3/1LA** | | | | | | | |
| *F*(22) = 29.199<br>*p* = <.001<br>$\eta_p^2$ = 0.57 | -2.83e-06 (2.53e-06) | *t*(22) = -11.1<br>*p* < .001<br>*d* = -2.32 | -1.14e-06 (1.54e-06) | *t*(22) = -3.01<br>*p* = .003<br>*d* = -0.63 | 2.07e-06 (1.28e-06 to 2.87e-06) | -0.32e-06 (1.39e-06) | 0.34e-06 (2.60e-06) | *t*(22) = 0.04<br>*p* = .068<br>*d* = 0.01 | -2.67e-08 ( -1.41e-06 to 1.36e-06) | 0.13 (0.02) | 0.13 (0.02) | *t*(22) = 1.37<br>*p* = .184<br>*d* = 0.29 | < -0.01 ( -0.01 to 0.00) |
| | | | | | | **FC4/1RA** | | | | | | | |
| *F*(22) = 15.653<br>*p* = <.001<br>$\eta_p^2$ = 0.42 | -2.73e-06 (2.50e-06) | *t*(22) = -11.0<br>*p* <.001<br>*d* = -2.29 | -1.23e-06 (1.97e-06) | *t*(22) = -3.11<br>*p* = .003<br>*d* = -0.65 | 1.88e-06 (8.95e-07 to 2.87e-06) | -0.28e-06 (1.49e-06) | 0.14e-06 (1.91e-06) | *t*(22) = -0.76<br>*p* = .453<br>*d* = -0.16 | 4.14e-07 ( -7.11e-07 to 1.54e-06) | 0.13 (0.02) | 0.12 (0.02) | *t*(22) = 1.26<br>*p* = 0.22<br>*d* = 0.26 | < -0.01 ( -0.02 to 0.00) |
| | | | | | | **C3/2LA** | | | | | | | |
| *F*(22) = 10.369<br>*p* = .004 | -2.548891e-06 2.409230e-06 | *t*(22) = -9.87<br>*p* < .001 | -7.514896e-07 2.224017e-06 | *t*(22) = -2.43<br>*p* = .01 | 1.77e-06 (6.31e-07 to | -0.46e-6 (1.31e-06) | -1.56 e-06 (3.33 e-06) | *t*(22) = 1.54 | -1.10e-06 ( -2.57e-06 to | 0.13 (0.02) | 0.12 (0.02) | *t*(22) = 2.36 | -0.01 ( -0.02 to -0.00) |

| | | | | | | | | | | | | | |
|---|---|---|---|---|---|---|---|---|---|---|---|---|---|
| $\eta_p^2$ = 0.32 | | *d* = -2.06 | | *d* = -0.51 | 2.92e-06) | | | *p* = .138<br><br>*d* = 0.32 | 3.81e-07) | | | *p* = .027<br><br>*d* = 0.49 | |
| **C4/2RA** | | | | | | | | | | | | | |
| *F*(22) = 14.161<br><br>*p* = .001<br><br>$\eta_p^2$ = 0.39 | -2.272809e-06 2.270693e-06 | *t*(22) = -10.00<br><br>*p* <.001<br><br>*d* = -2.09 | -8.622914e-07 1.934172e-06 | *t*(22) = -2.06<br><br>*p* = .026<br><br>*d* = -0.49 | 1.89e-06 (8.48e-07 to 2.93e-06) | -0.25e-6 (1.22e-06) | -1.41e-06 (2.22e-06) | *t*(22) = 2.16<br><br>*p* = .042<br><br>*d* = 0.45 | -1.16e-06 ( -2.28e-06 to -4.50e-08) | 0.13 (0.02) | 0.12 (0.02) | *t*(22) = 2.79<br><br>*p* = .011<br><br>*d* = 0.58 | -0.01 ( -0.02 to -0.00) |

*Note*. EEG = Electroencephalography. SD = Standard Deviation. BA = Bland-Altman. CI = Confidence Interval.

**Supplementary Table 3.**

*Comparison of connectivity measures between wet and dry EEG*

| Measure | n1 | n2 | Wet EEG | | Dry EEG | | Statistic | $p_{uncorr}$ | Effect size | Magnitude |
|---|---|---|---|---|---|---|---|---|---|---|
| | | | *M* | *SD* | *M* | *SD* | | | | |
| Alpha (8-13Hz) | | | | | | | | | | |
| aec | 32 | 32 | 0.13 | 0.05 | 0.09 | 0.03 | 413.00 | .004** | 0.55 | moderate |
| MST_degree | 32 | 32 | 0.36 | 0.04 | 0.37 | 0.08 | 239.00 | .647 | -0.11 | negligible |
| MST_Diam | 32 | 32 | 0.37 | 0.02 | 0.38 | 0.05 | 184.00 | .213 | -0.21 | small |
| MST_Kappa | 32 | 32 | 10.42 | 0.97 | 10.63 | 2.03 | 259.00 | .934 | -0.11 | negligible |
| MST_Leaf | 32 | 32 | 0.63 | 0.03 | 0.61 | 0.07 | 357.50 | .082 | 0.34 | small |
| MST_Teff | 32 | 32 | 0.20 | 0.04 | 0.22 | 0.04 | 197.00 | .217 | -0.26 | small |
| MST_Th | 32 | 32 | 0.42 | 0.02 | 0.40 | 0.03 | 416.00 | .004** | 0.58 | moderate |
| PLI | 32 | 32 | 0.14 | 0.04 | 0.13 | 0.04 | 456.00 | < .001*** | 0.70 | moderate |
| power | 32 | 32 | 0.19 | 0.07 | 0.11 | 0.05 | 527.00 | < .001*** | 1.59 | large |
| Beta (13-20/30Hz) | | | | | | | | | | |
| aec | 32 | 32 | 0.08 | 0.02 | 0.07 | 0.03 | 343.50 | .140 | 0.23 | small |
| MST_degree | 32 | 32 | 0.33 | 0.05 | 0.34 | 0.06 | 200.00 | .235 | -0.20 | negligible |
| MST_Diam | 32 | 32 | 0.39 | 0.03 | 0.40 | 0.04 | 210.50 | .888 | -0.03 | negligible |
| MST_Kappa | 32 | 32 | 9.75 | 0.99 | 10.10 | 1.44 | 205.00 | .278 | -0.22 | small |

| Measure | n1 | n2 | Wet EEG | | Dry EEG | | Statistic | $p_{uncorr}$ | Effect size | Magnitude |
|---|---|---|---|---|---|---|---|---|---|---|
| | | | ***M*** | ***SD*** | ***M*** | ***SD*** | | | | |
| MST_Leaf | 32 | 32 | 0.60 | 0.04 | 0.59 | 0.05 | 265.00 | .746 | 0.08 | negligible |
| MST_Teff | 32 | 32 | 0.21 | 0.03 | 0.21 | 0.04 | 263.00 | .993 | -0.05 | negligible |
| MST_Th | 32 | 32 | 0.41 | 0.02 | 0.40 | 0.03 | 323.00 | .278 | 0.24 | small |
| PLI | 32 | 32 | 0.10 | 0.01 | 0.10 | 0.01 | 415.00 | .004** | 0.54 | moderate |
| power | 32 | 32 | 0.22 | 0.07 | 0.12 | 0.06 | 513.00 | < .001*** | 1.42 | large |
| Delta (0.5-4Hz) | | | | | | | | | | |
| aec | 32 | 32 | 0.10 | 0.02 | 0.15 | 0.09 | 140.00 | .019* | -0.53 | moderate |
| MST_degree | 32 | 32 | 0.34 | 0.05 | 0.31 | 0.06 | 375.00 | .039* | 0.33 | small |
| MST_Diam | 32 | 32 | 0.39 | 0.03 | 0.41 | 0.04 | 98.00 | .006** | -0.55 | moderate |
| MST_Kappa | 32 | 32 | 9.96 | 0.96 | 9.44 | 1.17 | 392.00 | .016* | 0.35 | small |
| MST_Leaf | 32 | 32 | 0.60 | 0.04 | 0.57 | 0.04 | 440.50 | .001*** | 0.70 | moderate |
| MST_Teff | 32 | 32 | 0.21 | 0.04 | 0.22 | 0.03 | 185.00 | .144 | -0.27 | small |
| MST_Th | 32 | 32 | 0.41 | 0.03 | 0.39 | 0.02 | 438.00 | .001*** | 0.67 | moderate |
| PLI | 32 | 32 | 0.17 | 0.01 | 0.19 | 0.03 | 53.00 | < .001*** | -0.83 | large |
| power | 32 | 32 | 0.43 | 0.09 | 0.62 | 0.09 | 0.00 | < .001*** | -2.42 | large |
| Theta (4-8Hz) | | | | | | | | | | |
| aec | 32 | 32 | 0.10 | 0.03 | 0.11 | 0.04 | 189.00 | .166 | -0.31 | small |

| Measure | n1 | n2 | Wet EEG | | Dry EEG | | Statistic | $p_{uncorr}$ | Effect size | Magnitude |
|---|---|---|---|---|---|---|---|---|---|---|
| | | | ***M*** | ***SD*** | ***M*** | ***SD*** | | | | |
| MST_degree | 32 | 32 | 0.34 | 0.03 | 0.34 | 0.07 | 276.50 | .822 | -0.02 | negligible |
| MST_Diam | 32 | 32 | 0.39 | 0.03 | 0.40 | 0.04 | 205.50 | .278 | -0.21 | small |
| MST_Kappa | 32 | 32 | 10.00 | 0.71 | 9.99 | 1.59 | 284.00 | .719 | 0.01 | negligible |
| MST_Leaf | 32 | 32 | 0.61 | 0.03 | 0.58 | 0.05 | 369.50 | .050* | 0.39 | small |
| MST_Teff | 32 | 32 | 0.20 | 0.04 | 0.22 | 0.04 | 201.00 | .243 | -0.24 | small |
| MST_Th | 32 | 32 | 0.41 | 0.02 | 0.40 | 0.02 | 415.00 | .004** | 0.57 | moderate |
| PLI | 32 | 32 | 0.14 | 0.01 | 0.15 | 0.01 | 195.00 | .200 | -0.24 | small |
| power | 32 | 32 | 0.16 | 0.05 | 0.15 | 0.04 | 335.00 | .190 | 0.27 | small |

*Notes.* aec = amplitude envelope correlation. MST = minimum spanning tree. PLI = phase lag index. The statistic is derived with a paired Wilcoxon *t*-test. Effect size is estimated with Cohen's *d*. * $p < .05$, ** $p < .01$, *** $p < .001$.
